# *CDKN2A* as transcriptomic marker for muscle-invasive bladder cancer risk stratification and therapy decision-making

**DOI:** 10.1038/s41598-018-32569-x

**Published:** 2018-09-26

**Authors:** Thomas S. Worst, Cleo-Aron Weis, Robert Stöhr, Simone Bertz, Markus Eckstein, Wolfgang Otto, Johannes Breyer, Arndt Hartmann, Christian Bolenz, Ralph M. Wirtz, Philipp Erben

**Affiliations:** 1 Department of Urology, University Medical Center Mannheim, Theodor-Kutzer-Ufer 1-3, 68167 Mannheim, Germany; 2 Institute of Pathology, University Medical Center Mannheim, Theodor-Kutzer-Ufer 1-3, 68167 Mannheim, Germany; 3 Institute of Pathology, University of Erlangen-Nuremberg, Krankenhausstraße 8-10, 91054 Erlangen, Germany; 4 Department of Urology, University of Regensburg, Landshuter Straße 65, 93053 Regensburg, Germany; 5 Department of Urology, University of Ulm, Prittwitzstraße 43, 89075 Ulm, Germany; 6 STRATIFYER Molecular Pathology GmbH, Werthmannstraße 1, 50935 Cologne, Germany; 7 Institute of Pathology at the St Elisabeth Hospital Köln-Hohenlind, Werthmannstraße 1, 50935 Cologne, Germany

## Abstract

Deletions of the cell cycle control gene *CDKN2A* are described as progression markers of non-muscle invasive bladder cancer and to be associated with fibroblast growth factor 3 (*FGFR3*) mutations. The prognostic role of *CDKN2A* RNA expression in muscle invasive bladder cancer (MIBC) is under discussion. In 80 MIBC patients (m/f 60/20) who underwent radical cystectomy the expression of *CDKN2A* and *FGFR3* was examined with qRT-PCR (test cohort). The MDA cohort (n = 57) and the TCGA cohort (n = 365) served for validation. The expression of drug target genes and TCGA molecular subtypes was correlated with *CDKN2A* expression. In the test cohort *CDKN2A*^high^ patients (n = 8; 10.0%) had a significantly shorter recurrence-free (p = 0.018) and disease-specific (p = 0.006) survival compared to the rest of the cohort. A similar stratification was seen in the validation cohorts (*CDKN2A*^high^: n = 7, 12.3%, p = 0.001; n = 46, 12.6%, p = 0.011). In the TCGA cohort these patients had a comparably low expression of drug target genes. The expression of *CDKN2A* significantly differed among TGCA molecular subtypes. 71.7% of *CDKN2A*^high^ were TCGA basal squamous tumours but also show divergent molecular features compared to this group. In summary *CDKN2A* RNA expression-based risk stratification of MIBC allows the identification of a *CDKN2A*^high^ poor prognosis group with low expression of drug target genes.

## Introduction

For several decades radical cystectomy (RC) is the standard therapy of muscle invasive bladder cancer (MIBC). Yet, due to a high recurrence rate, 5-year overall survival (OS) of patients with locally advanced tumours is only around 50%^1^. In the hospital routine decisions on adjuvant, neoadjuvant and palliative medication still mainly rely on clinical parameters. Though deemed crucial in terms of risk stratification and identification of patients in need for a more aggressive treatment, molecular profiling for individual therapy decision-making is still in it’s infancy in MIBC^2,3^. Furthermore, expression data can give valuable information about drug target gene expression^4–6^.

In the light of bladder cancer initiation several frequent genetic aberrations have been identified. Papillary/non muscle-invasive and non-papillary/muscle-invasive bladder cancer are typically seen as two different molecular entities^7^. In both groups alterations of “forerunner genes” are seen as an initial event. Whilst in papillary tumours, genetic alterations are mainly restricted to these genes, high risk NMIBC and MIBC often show alterations of major tumour suppressor genes as *RB1* or *TP53*^8,9^.

Loss of heterozygosity (LOH) in the 9p region is one of this typical early events in the formation of bladder cancer and frequently occurs in non-invasive precursor lesions like hyperplasia, dysplasia or carcinoma *in situ*^10–13^. One of the genes found in this region is *CDKN2A*, which codes for the cell cycle control protein p16. LOH of *CDKN2A* and decreased expression of the p16 protein are mainly described as a predictor of progression in non muscle-invasive bladder cancer (NMIBC)^14^. Homozygous deletion of *CDKN2A*, is also associated with muscle invasion in *FGFR3*-mutated (fibroblast growth factor receptor 3) tumours^15^.

On the protein level a meta-analysis^16^, including data from 17 immunohistochemistry studies with 1032 subjects, investigated the p16 expression in various disease stages and found a significant association between a low expression of p16 and recurrence-free survival in patients with all stages of bladder cancer. When stratifying for T stages this correlation was markedly stronger for NMIBC, but was not found for MIBC (≥T2). The same was found for progression-free survival (PFS). The authors concluded that the p16 expression is affected by clinicopathologic stage and its relevance is mainly to be seen in NMIBC.

Another study found altered p16 protein expression, defined as either no expression of p16 or a very strong p16 expression, to be associated with a worse outcome of MIBC^17^. These results support a more complex role of *CDKN2A* and the p16 protein in MIBC.

We therefore aimed to stratify patients with MBIC according to their *CDKN2A* expression. Since immunohistochemistry is limited in case of quantification and sample comparison, RNA-based methods like qRT-PCR or next generation sequencing are robust alternatives for quantification and stratification of gene expression. The value of *CDKN2A* mRNA expression has not been systematically investigated in MIBC, yet, but qRT-PCR has already proved to be a valuable tool to determine *CDKN2A* copy number status^18^. The *CDKN2A* RNA-expression-based risk stratification was validated in the MDA and the TCGA cohort. Furthermore we reanalysed TCGA data to reveal correlations of *CDKN2A* with drug target gene expression and molecular subtypes.

## Results

### *CDKN2A* RNA expression allows risk stratification of MIBC patients

When stratifying for disease-specific death using the partition test, the test cohort of 80 patients with MIBC could be divided into two groups with different *CDKN2A* expression (*CDKN2A*^high^ with n = 8, 10.0%; *CDKN2A*^low^ with n = 72, 90.0%). Clinicopathologic data did not differ significantly between these groups (Table 1

**Table 1:** Patient characteristics of the test cohort.

parameter	total (n = 80)	*CDKN2A*		p-value (Chi^2^)
		low (n = 72)	high (n = 8)	
Male Female	60 (75.0%) 20 (25.0%)	55 (76.4%) 17 (23.6%)	5 (62.5%) 3 (37.5%)	p = 0.389
Age	66 (46–93)	66 (46–85)	72 (54–93)	t-test p = 0.081
T2 T3 T4	19 (23.8%) 47 (58.8%) 14 (16.5%)	18 (25.0%) 42 (58.3%) 12 (16.7%)	1 (12.5%) 5 (62.5%) 2 (25.5%)	p = 0.677
N0 N1 Nx	48 (60.0%) 32 (40.0%) —	44 (61.1%) 28 (38.9%) —	4 (50.0%) 4 (50.0%) —	p = 0.543

).

**Figure 1:**
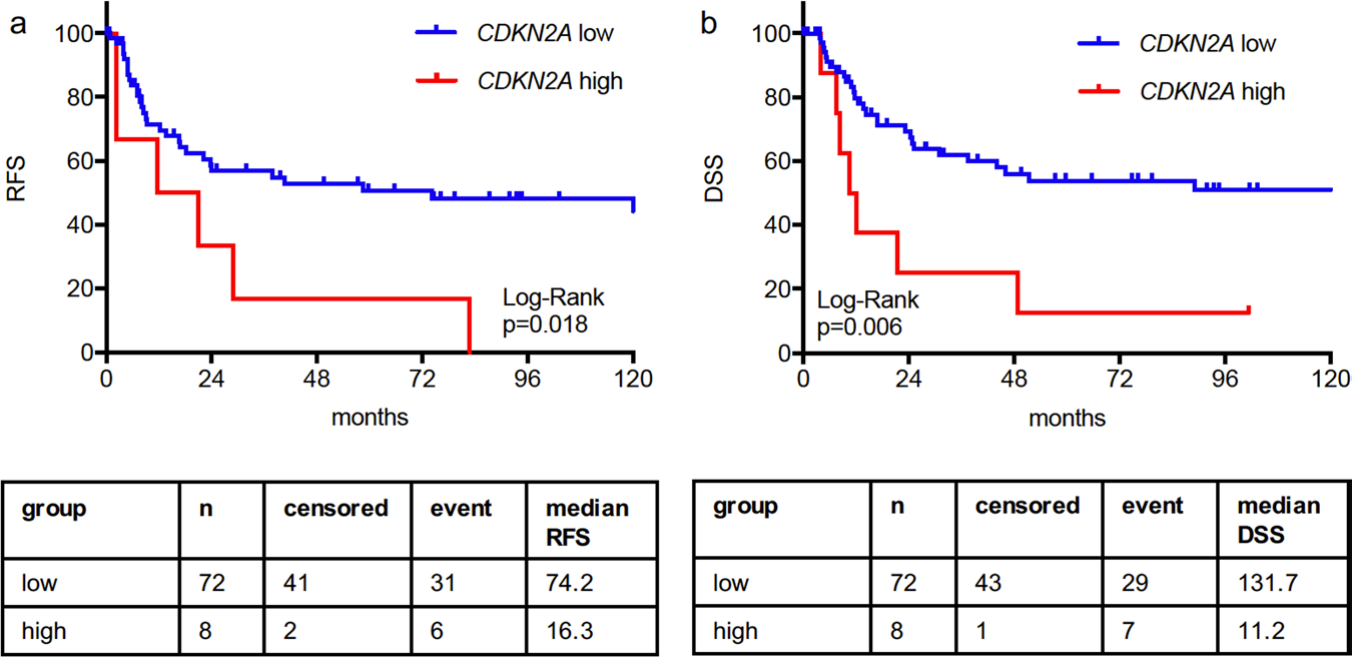
Stratification of the test cohort (n = 80) for *CDKN2A* expression identified a subgroup of 8/80 (10.0%) patients with the highest *CDKN2A* expression who had a much worse RFS (**a**) and DSS (**b**) compared to the rest of the cohort.

Kaplan-Meier analysis for recurrence-free survival (RFS, Fig. 1a) and disease-specific survival (DSS, Fig. 1b) showed significant differences (p = 0.018 and p = 0.006) between these groups, with *CDKN2A*^high^ having a worse prognosis (median RFS 16.3 months and median DSS 11.2 months) compared to patients with *CDKN2A*^low^ tumours (median RFS 74.2 months and median DSS 131.7 months).

**Figure 2:**
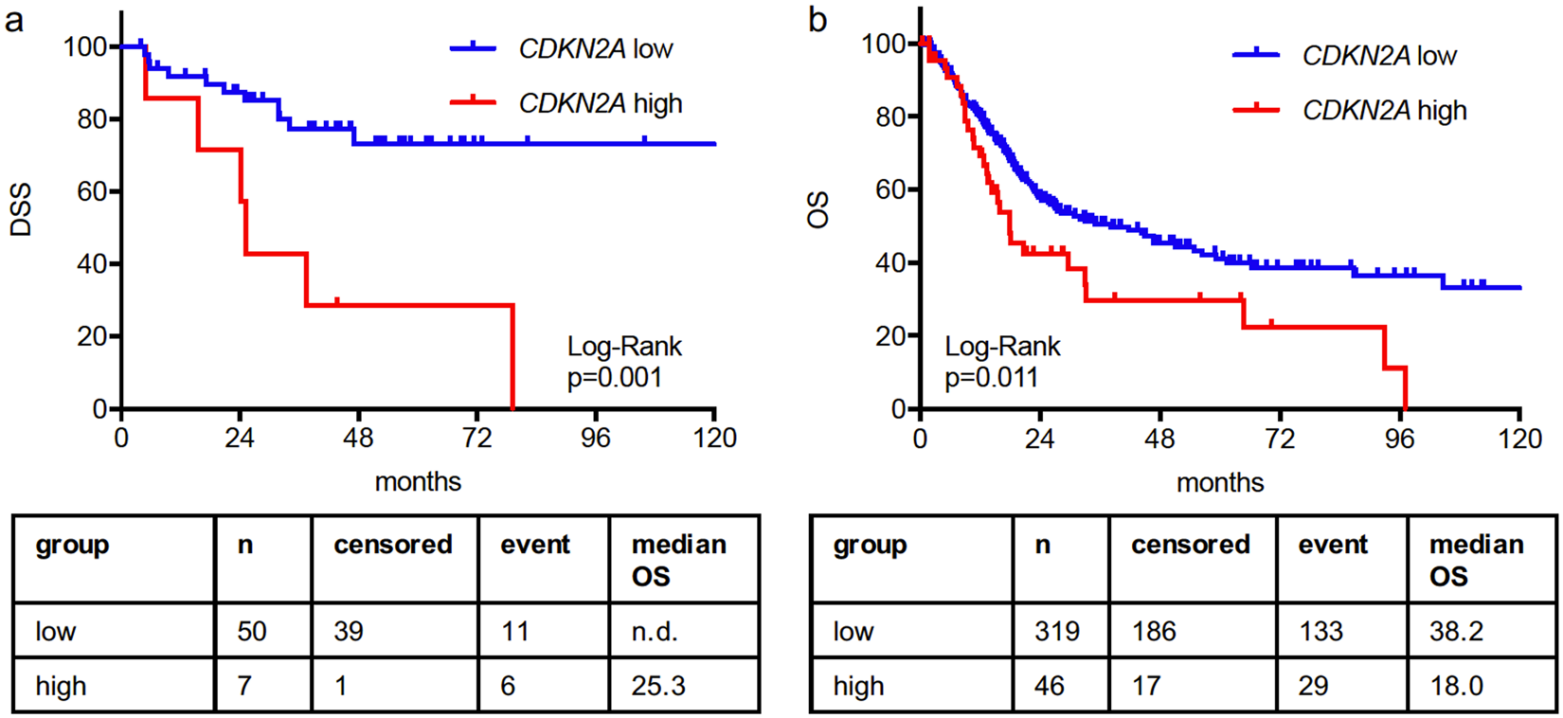
Validation in (**a**) the MDA cohort and (**b**) the TCGA cohort confirmed the poor prognosis of a similar proportion of patients (**a**: 12,3% and **b**: 12,6%) with the highest *CDKN2A* expression. (n.d. = not defined).

By using the partition test in the MDA cohort of 57 bladder cancer patients a similar cut-off for *CDKN2A* expression could be defined. As in the test cohort, those patients with the highest *CDKN2A* expression (n = 7; 12.3%) had a worse prognosis (p = 0.001; median DSS 25.3 months, for *CDKN2A* median DSS was not reached; Fig. 2a).

When applying the partition test to the TCGA cohort, again a small group of patients with the highest *CDKN2A* expression (n = 46; 12.6%) with a poor prognosis could be identified (p = 0.011; Fig. 2b). The median overall survival (OS) of *CDKN2A*^high^ was 18.0 months, compared to 38.2 months in the *CDKN2A*^*l*ow^ group (n = 319; 87.4%). Clinicopathologic data did not differ significantly between the *CDKN2A* expression groups in the MDA and the TCGA cohort (Table 2 and Table 3

**Table 2:** Patient characteristics of the MDA cohort.

parameter	total (n = 57)	*CDKN2A*		p-value (Chi^2^)
		low (n = 50)	high (n = 7)	
Male Female	49 (85.9%) 8 (14.1%)	43 (86.0%) 7 (14.0%)	6 (85.7%) 1 (14.3%)	p = 0.984
Age	66 (41–89)	66 (41–89)	61 (41–85)	t-test p = 0.302
T1 T2 T3 T4	2 (3.5%) 12 (21.1%) 35 (61.4%) 8 (14.0%)	1 (2.0%) 12 (24.0%) 31 (62.0%) 4 (8.0%)	1 (14.2%) 0 (0.0%) 4 (57.1%) 2 (28.6%)	p = 0.086
N0 N1 N2	22 (38.6%) 9 (15.8%) 26 (45.6%)	19 (38.0%) 8 (16.0%) 23 (46.0%)	3 (42.9%) 1 (14.2%) 3 (42.9%)	p = 0.969

**Table 3:** Patient characteristics of the TGCA cohort.

parameter	total (n = 365)	*CDKN2A*		p-value (Chi^2^)
		low (n = 319)	high (n = 46)	
Male Female	269 (73.7%) 96 (26.3%)	239 (74.9%) 80 (25.1%)	30 (65.2%) 16 (34.8%)	p = 0.162
Age	68 (34–90)	68 (34–90)	68 (44–90)	t-test p = 0.989
T2 T3 T4	118 (32.3%) 190 (52.1%) 57 (15.6%)	104 (32.6%) 164 (51.4%) 51 (16.0%)	14 (30.4%) 26 (56.6%) 6 (13.0%)	p = 0.785
N0 N1 Nx	217 (59.5%) 125 (34.2%) 23 (6.3%)	191 (59.9%) 108 (33.9%) 20 (6.2%)	26 (56.6%) 17 (36.9%) 3 (6.5%)	p = 0.908
Neoadjuvant treatment				p = 0.249
Yes	9 (2.47%)	9 (2.8%)	0 (0.0%)	
No	356 (97.5%)	310 (97.2%)	46 (100.0%)	
Adjuvant chemotherapy				p = 0.0582
Yes	62 (17.0%)	58 (18.2%)	4 (8.7%)	
No	158 (43.3%)	131 (41.1%)	27 (58.7%)	
NA	145 (39.7%)	130 (40.7%)	15 (32.6%)	
Adjuvant radiotherapy				p = 0.167
Yes	7 (1.9%)	7 (2.2%)	0 (0.0%)	
No	227 (62.2%)	193 (60.5%)	34 (73.9%)	
NA	131 (35.9%)	119 (37.3%)	12 (26.1%)	

).

Further dissection of the bigger group of CDKN2A^low^ tumours in the test cohort and the TCGA cohort did not result in similar subgroup sizes, but yet resulted in different cut-offs with significant differences in prognosis, with patients with an intermediate expression of CDKN2A having a better prognosis as those with a low expression (Supplementary Figure 1).

### Expression of drug target genes in dependence on *CDKN2A* expression

**Figure 3:**
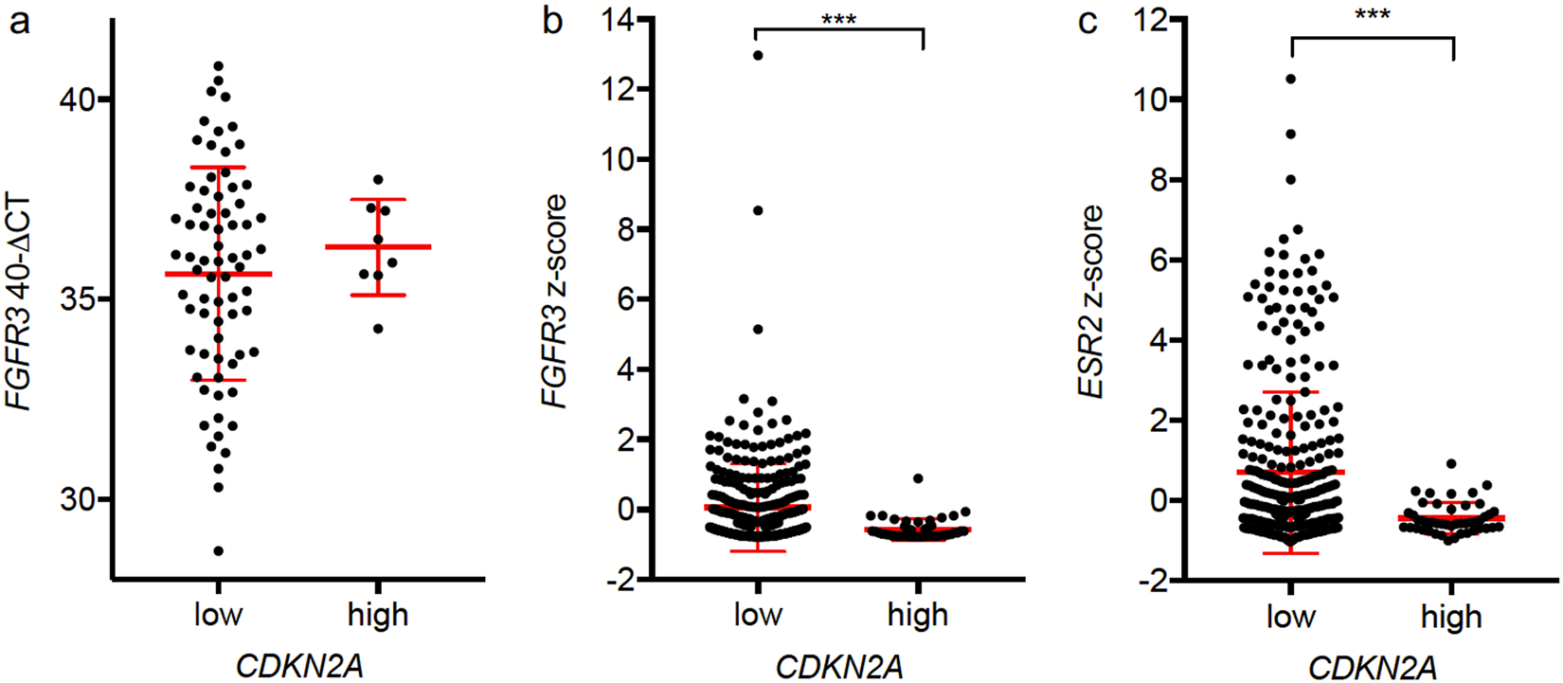
(**a**) In the test cohort there was no difference in the *FGFR3* expression in the *CDKN2A* expression groups. (**b**) In the TCGA cohort *FGFR3* was significantly lower expressed in *CDKN2A*^*high*^ tumours. *CDKN2A*^low^ tumours had also a higher expression of *ESR2* (**c**). (*p < 0.05; **p < 0.01; ***p < 0.001).

In the TCGA cohort there was a negative correlation between *CDKN2A* and *FGFR3* in all MIBC (ρ = −0.406; p < 0.001). In the test cohort there was also a trend towards a negative correlation between *FGFR3* and *CDKN2A* (ρ = −0.217, p = 0.053). Yet, inter-group comparison did not show a significant difference in *FGFR3* expression between *CDKN2A*^low^ and *CDKN2A*^high^ tumours (p = 0493; Fig. 3a). In the TCGA cohort the *FGFR3* expression differed significantly between the *CDKN2A* expression groups (p < 0.001; Fig. 3b).

Stratification for *CDKN2A* expression also showed a significantly lower expression of *ESR2* in *CDKN2A*^high^ tumours (p < 0.001, Fig. 3c). For the other tested drug target genes (*AR, ESR1, ERBB2, PDCD1, CD274* and *CTLA4*) no significant differences in the two *CDKN2A* expression groups were seen. Over all MIBC patients from the TCGA cohort *AR* was negatively correlated with *CDKN2A* expression (ρ = −0.183; p = 0.004) and *PDCD1*, *CD274* and *CTLA4* were positively correlated with *CDKN2A* expression (ρ = 0.176; p < 0.001; ρ = 0.327; p < 0.001; ρ = 0.171; p < 0.001). Detailed results are given in Supplementary Table 1.

### *CDKN2A* CNV status, downstream target expression and molecular subtypes

In the TCGA cohort patients with *CDKN2A*^low^ expression frequently had *CDKN2A* deletions (38.9%% homozygous deletion, heterozygous deletions: 26.0%) in comparison to more balanced genotypes in the *CDKN2A*^high^ group (−1: 21.7%, balanced: 34.8%, +1: 43.5%, Supplementary Figure 2a). Chi^2^ test proved this difference to by significant (p < 0.001). Vice versa, upon stratification of *CDKN2A* expression for *CDKN2A* CNV there was also a significant difference between CNV groups (Kruskal-Wallis p < 0.001, Supplementary Figure 2b).

Supplementary Figure 3a illustrates the *CDKN2A* expression in the *CDKN2A* expression groups of the TCGA cohort. *CDK4*, despite being negatively regulated by *CDKN2A*, showed a slightly, but not significantly higher expression in *CDKN2A*^*high*^ (p < 0.139; Supplementary Figure 3b). *RB1*, which is regulated by *CDK4*, showed a significantly lower expression in *CDKN2A*^*high*^ (p < 0.001, Supplementary Figure 3c) and the downstream transcription factor gene *E2F3* was significantly higher expressed in *CDKN2A*^high^ tumours (p < 0.001, Supplementary Figure 3d). In the complete cohort a positive correlation was observed between *CDKN2A* and *CDK4* gene expression (ρ = 0.222, p < 0.001) and *E2F3* gene expression (ρ = 0.212, p < 0.001) and a negative correlation between *CDKN2A* and *RB1* ρ = −0.479 (p < 0.001).

**Figure 4:**
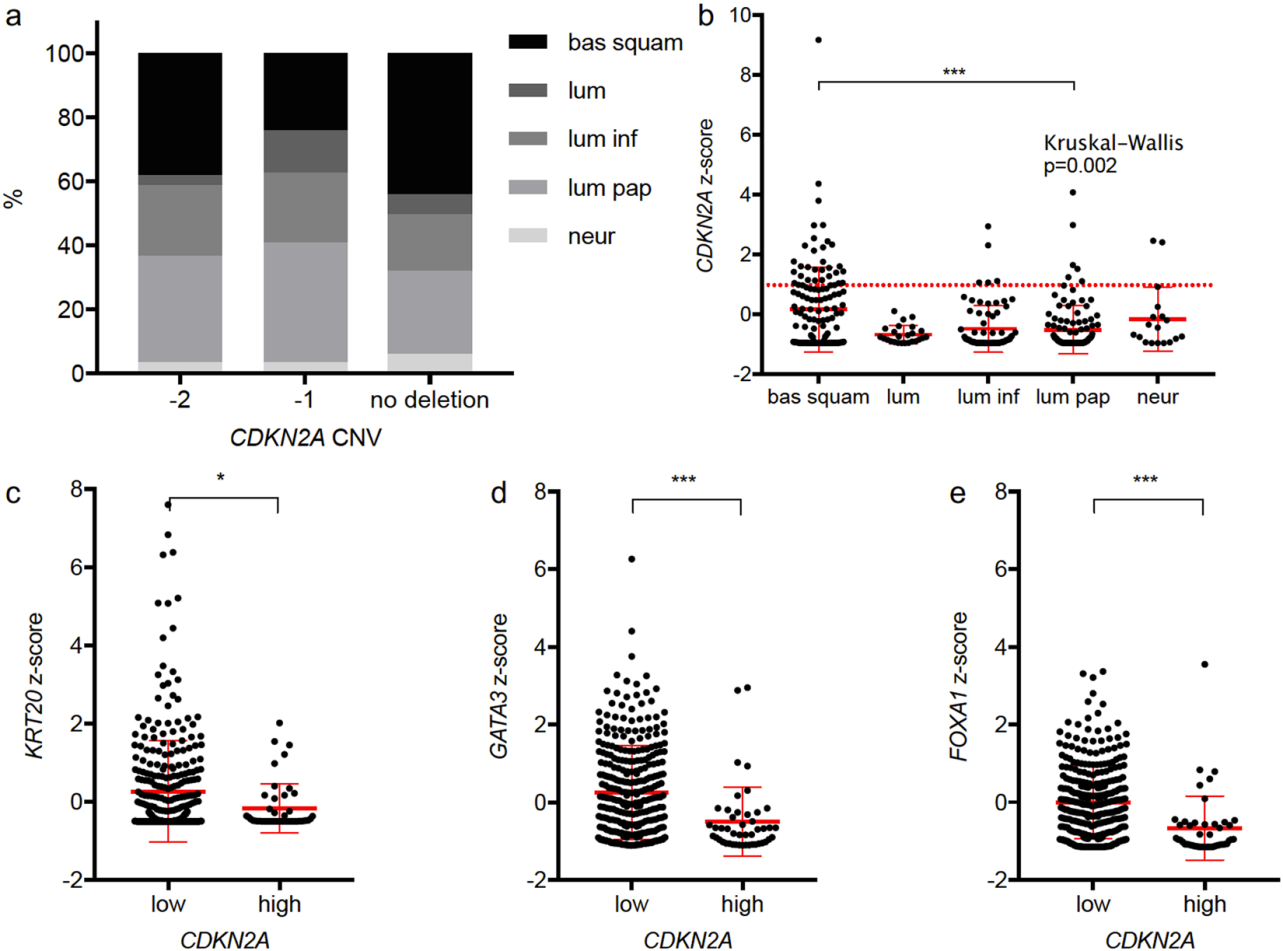
(**a**) In the TCGA cohort the distribution of TCGA RNA-expression subtypes significantly differs according to *CDKN2A* copy number status (Chi^2^ 0.016). (**b**) Vice versa there were significant differences in the *CDKN2A* expression in these subtypes (bas squam = basal squamous, lum = luminal, lum inf = luminal infiltrated, lum pap = luminal papillary, neur = neuronal). Of the analyzed, typically subtype defining, genes, *KRT20*, *GATA3* and *FOXA1* showed a differing expression in the *CDKN2A* expression groups (**c**–**e**). (*p < 0.05; **p < 0.01; ***p < 0.001).

When correlated with copy number status, there was a significant difference in the distribution of updated TCGA subtypes^19^ (Chi^2^ p = 0.016; Table 4 and Fig. 4a). Tumours with basal squamous and neuronal expression phenotype were overrepresented in the group of tumours with no deletion. Luminal tumours mainly had a homozygous deletion. Tumours from the basal squamous group on average showed the highest *CDKN2A* expression and tumours from the luminal group had a comparably low *CDKN2A* expression. Over all groups Kruskal-Wallis test showed a significantly different distribution, with 33 of 46 tumours (71.7%) in the *CDKN2A*^high^ group being classified as basal squamous (Fig. 4b).

**Table 4:** Chi^2^-square test showed significant difference in the distribution of TCGA subtypes according to copy number status.

Copy number status	Subtypes					p-value (Chi^2^)
	Basal squamous	Luminal	Luminal infiltrated	Luminal papillary	Neuronal	
−2	47	4	27	41	4	p = 0.016
−1	22	12	20	34	3	
no deletion	65	9	26	38	9

When looking at specific genes, typically determining RNA expression subtypes, *GATA3* (ρ = −0.162; p = 0.002) and *FOXA1* (ρ = −0.299; p < 0.001) showed an inverse correlation with the *CDKN2A* expression. Both genes also showed a significantly lower expression in *CDKN2A*^high^ tumours compared to *CDKN2A*^low^ tumours (both p < 0.001; Fig. 4d,e). Furthermore *KRT20* was significantly lower expressed in CDKN2A^high^ tumours (p = 0.028; Fig. 4c). Detailed results are given in Supplementary Table 2.

## Discussion

Deletions of *CDKN2A* and the underexpression of p16, the protein coded by *CDKN2A*, are well-investigated molecular risk factors for tumour progression in NMIBC. Based on this, one could conclude that deletion or underexpression of *CDKN2A*/p16 is also an indicator of increased aggressiveness and worse prognosis in MIBC. Yet, some data point to a more complex situation in MIBC. For instance, gene expression studies have shown distinct RNA expression patterns for Ta tumours and MIBC, with T1 tumours showing either one or the other signature^20^. To more deeply investigate the role of *CDKN2A* expression in tumour prognosis and its association with drug target genes like *FGFR3*, we performed qRT-PCR expression profiling and reanalysis of existing *CDKN2A* and *FGFR3* RNA expression data of MIBC after RC. Compared to immunohistochemistry studies of p16, RNA-testing with qRT-PCR has the advantages of a higher dynamic width and a higher sensitivity. Furthermore qRT-PCR testing allows an observer-independent interpretation of quantifiably results.

In the three analyzed cohorts the groups with the highest expression of *CDKN2A* (10.0–12.6% of the examined patients) were identified to have a worse prognosis compared to the remaining patients. This is controversial to previous assumptions derived from findings in NMIBC, where deletion of *CDKN2A*, typically going along with a lowered expression, is deemed as a marker for poor prognosis^2,21,22^.

However, already in 2004 it was shown that both a low and a high expression of the p16 protein can be a predictor of worse outcome after RC^17^. The overall prevalence of altered p16 protein expression was 54% of the analyzed tumours. The results of last-mentioned study show that both high and low expression of *CDKN2A* and p16, respectively, are associated with a worse outcome of MIBC patients. Though we could not find similar cut-off values in the different cohorts to distinguish between patients with low and intermediate expression of CDKN2A in the present study, patients with an intermediate expression seem to have the best prognosis, pointing to a diverse role of *CDKN2A* as prognosis marker in MIBC.

Functionally it is well known that impaired function or expression of p16, either due to *CDKN2A* deletion, mutation or hypermethylation, leads to cell cycle deregulation via overactivation of CDK4 and CDK6, which results in hyperphosphorylation of retinoblastoma protein (RB), the protein product of *RB1*. The subsequent liberation of E2F transcription factor family members mediates changes in gene expression, promoting the transition from G1 to S phase^23^. Besides this mechanism, loss of *RB1* also results in tumour formation and progression^24^.

This close relation between *CDKN2A*/p16 and RB has been repeatedly described in urinary bladder cancer^25,26^. Yet, this does not intuitively explain why those MIBC with the highest *CDKN2A* expression show a poor prognosis. Sjödahl *et al*. reported two different genomic circuits operative in urothelial carcinomas: one defined by high *FGFR3* and *CCND1* expression, low *CDKN2A* expression, often associated with *CDKN2A* loss and the other one defined by *E2F3* amplifications and overexpression, *RB1* deletions and low expression and high *CDKN2A*/p16 expression^27^. Whilst the first circuit is mainly found in tumours termed urobasal A and urobasal B, the latter circuit was mainly associated with genomically unstable tumours^28,29^. These tumours also on the protein level typically showed no or low expression of KRT5 and KRT14, aberrant expression of KRT20 and a low expression of EGFR, but a high expression of ERBB2. According to this immunohistochemistry-based classification, genetically unstable tumours had a worse DSS compared to urobasal tumours, but better than squamous cancer cell-like tumours in a mixed population of NMIBC and MIBC^27^. Recent work from our own group has also shown high *CDKN2A* expression to be associated with shorter progress-free survival in T1 urothelial carcinoma^30^. The group of *CDKN2A*^high^ MIBC consistently identified in a proportion between 10.0 and 12.6% in all three datasets analyzed in the present study therefore might reflect a subgroup of genomically instable tumours, which account for 21.5% of advanced bladder cancers as described by Sjödahl *et al*.^29^. The reported overexpression of *CDKN2A* in genomically unstable tumours could be a sign of an in vain countermeasure to reduce cell cycle activity, which is deregulated due to other molecular aberrations.

Controversial to an association with the genomically unstable subtype is the fact, that 71,7% of the tumours with *CDKN2A*^high^ from the TCGA cohort are termed as basal squamous according to the 2017 TCGA publication^19^. Yet, this group (35% of MIBC in the TCGA cohort), also comprises 41% of tumors with *CDKN2A* deep deletions, meaning that *CDKN2A*^high^ tumours, which do not show any homozygous deletion of *CDKN2A*, are in part not a representative, but a highly selected subgroup of basal squamous tumours. In line with this and unlike reported for basal squamous tumors in the TCGA publication, *CDKN2A*^high^ tumours also do not show an elevation of *PDCD1*, *CD274* and *CTLA4* expression.

With regard to drug target gene expression, *CDKN2A* expression showed a negative correlation with *FGFR3* expression in the TCGA cohort and a trend towards a negative correlation in the test cohort. The TCGA publication from 2014 proposed a correlation between *CDKN2A* deletion or underexpression and activating mutations of *FGFR3* or *FGFR3* overexpression^4^. They also reported an inverse correlation between *CDKN2A* and *FGFR3* RNA expression. According to their mutational data they proposed three subtypes of bladder cancer: (A) *focally amplified*, (B) *papillary CDKN2A-deficient and FGFR3-mutant* and (C) *TP53/cell-cycle-mutant*. The study of Rebouissou and colleagues confirmed a high incidence of *CDKN2A* deletions (hemizygous 23.7%, homozygous 17.5%) and *FGFR3* mutations (62.1%) in NMIBC^15^. In MIBC the rates of *CDKN2A* deletions were even higher (hemizygous 27.9%, homozygous 22.5%). Both in NMIBC and in MIBC there was a significant coincidence of *CDKN2A* deletions and activating mutations of *FGFR3* and NMIBC tumours with this feature had an increased progression rate.

Due to their low *FGFR3* expression, patients with CDKN2A^high^ tumours presumably do not seem to be suitable candidates for a therapy targeting FGFR3, whilst patients with a high *FGFR3* expression might benefit from such an approach. The tyrosine kinases inhibitor Pazopanib is already approved for the treatment of advanced or metastatic kidney cancer and certain sarcoma entities, but there is only limited data about its application in MIBC: A small phase II trial on 19 unselected patients with metastatic bladder cancer reported a median PFS of only 1.9 months^31^. Another phase II study of 41 unselected patients with metastatic bladder cancer after failure of chemotherapy reported an overall initial response rate of 17%, but PFS and OS were poor^32^. However, there were also two patients with sustained long-term response. The RNA expression of *FGFR3* in these tumours is not reported in the trial. Another group reported a case of a woman with a metastatic bladder cancer carrying an activating *FGFR3* mutation^33^. This patient showed a durable remission of more than 6 months upon treatment with Pazopanib. *In vitro* results also suggest a synergistic effect of Pazopanib with Docetaxel in the treatment of bladder cancer cells^34^, pointing to a potential role of Pazopanib in combination therapy of cases with a suitable molecular profile. Besides Pazopanib, several other substances targeting FGFRs are currently under investigation^35^ and AZ12908010, AZD4547, PD173074, TKI-258/Dovitinib, SU5402 and BGJ-398 showed promising results *in vitro*^36–39^. Yet, clinical data is scarce and partially controversial: By systemic administration of Dovitinib biologically active concentrations could be consistently achieved in 13 patients with NMIBC^40^. However, long-term administration was not possible due to frequent toxicities. In another study Dovitinib showed a better tolerability but the antineoplastic effect in patients with FGFR3-mutated and FGFR3 wild type urothelial bladder cancer was poor^41^. For AZD4547 a case of long term response is described^42^. BGJ-398 showed an overall response rate of 36% in patients with pretreated advanced or metastatic urothelial carcinoma and was well tolerated^43^.

Besides a low *FGFR3* expression, *CDKN2A*^*high*^ tumours also showed a low *ESR2* expression and *AR* was negatively correlated with *CDKN2A*. *CDKN2A* expression was positively correlated with *PDCD1*, *CD274* and *CTLA4* expression. Yet, this did not result in a differential expression in *CDKN2A*^*high*^ tumours. In general the expression of the tested drug target genes was rather low in *CDKN2A*^*high*^ tumours. Therefore they may represent a high risk population both in terms of prognosis and limited treatment options.

Correlation of *CDKN2A* with the downstream markers *CDK4*, *RB1* and *E2F3* in the TCGA cohort also point to a more complex role of *CDKN2A* in the biology of MIBC: Unlike to be assumed by the known mechanism of *CDKN2A*-*CDK4* interaction, with *CDKN2A* typically deactivating *CDK4*, the expression of both genes is not inversely but positively correlated. Furthermore, the tumour suppressor *RB1*, which is the subsequent gene in this signaling cascade^44^, is significantly downregulated upon increasing *CDKN2A* expression, indicating a more active cell cycle despite a high *CDKN2A* expression. Since the P16 protein mainly functionally regulates downstream targets via binding of CDK4 and CDK6, preventing them from interaction with cyclin D, which then results in reduced phosphorylation of RB1, phosphorylation data of RB1 would offer a more precise information about the pathway activity downstream of P16. Yet, there are currently no larger datasets analyzing RB1 phosphorylation status in bladder cancer.

The 2014 TCGA publication, comprising data from 131 MIBC, described four different subtypes based on RNA expression data^4^. These subtypes are mainly determined by the expression of luminal cytokeratins *KRT8* and *KRT18*, basal cytokeratins *KRT5*, *KRT6A*, *KRT6B*, *KRT6C*, *KRT15*, the transcription factors *GATA3* and *FOXA1* and uroplakins. The updated 2017 publication^19^ suggests five molecular subtypes and implemented elements from other subtyping approaches^8,9,45^. We correlated the expression of *CDKN2A* with the aforementioned subtype determining genes. In the TCGA cohort there was a negative correlation with *GATA3* and *FOXA1* (ρ = −0.162 and −0.299), which are urothelial differentiation markers. For both genes also a significantly lower expression was seen in *CDKN2A*^*high*^ tumours. And also *KRT20*, typically associated with luminal tumours, showed a lower expression in this group. Fitting to this TCGA basal squamous tumours have the highest *CDKN2A* expression, whilst tumours from the luminal TCGA subtype group had an exclusively low expression.

Our results are limited by the fact that both our *CDKN2A* PCR assay and the RNA expression analysis in the MDA and the TCGA cohort do not discriminate between the *CDKN2A* transcripts. Therefore further analyses substratifying between the transcripts for p16 and p14 could add valuable information.

Furthermore the three analyzed cohorts are not directly comparable due to the different techniques (qRT-PCR in the test cohort, RNA expression microarray in the MDA cohort and RNAseq in the TCGA cohort) used for quantification and different data normalization protocols.

Taken together the *CDKN2A* RNA expression does not present as a continuum. On the one hand this indicates that quantifiably tools can be helpful to accurately deduce prognosis from *CDKN2A* RNA expression. On the other hand this also reflects a more complex biology behind the variation of *CDKN2A* expression in MIBC. In line with this, the expression of *CDKN2A* and p16 differs among molecular subtypes.

With the chosen approach we were able to identify a subgroup of patients with high *CDKN2A* expression with poor prognosis and comparably low expression of drug target genes. These tumours seem to partly correspond to both the basal squamous subtype described in the 2017 TCGA classification^19^ and the genomically unstable tumours described by Sjödahl *et al*.^29^. *CDKN2A* therefore might be a valuable component in the molecular risk stratification of MIBC and a potential indicator for targeted therapy decision-making.

## Methods

### Study population, RNA isolation and qRT-PCR

The test cohort consisted of 80 patients (mean age 66 years, range 46–93 years) with MIBC who underwent radical cystectomy at the Mannheim University Hospital Center between January 1998 and December 2006. Clinical and pathological data were retrospectively obtained from medical records (ethics approval 2016-814R-MA of the medical ethics committee II of the medical faculty Mannheim of the University of Heidelberg).

RNA extraction was performed as described before^46^. 10 μm sections from FFPE tissue samples were used for RNA extraction with a commercially available bead-based extraction method (XTRACT kit; STRATIFYER Molecular Pathology GmbH, Cologne, Germany). RNA was eluted with 100 μl elution buffer and stored at −80 °C until used.

The RNA expression of *CDKN2A* and *FGFR3* was determined in relation to the housekeeping gene calmodulin 2 (*CALM2*) using 1-step qRT-PCR with validated TaqMan gene expression assays (STRATIFYER catalogue numbers: *CDKN2A*: MP672, *FGFR3*: MP599 and *CALM2*: MP501). Primers and labeled hydrolysis probes were selected using Primer Express® Software (Applied Biosystems/Life Technologies, Karlsruhe, Germany) and were controlled for single nucleotide polymorphisms. All primers, probes and amplicons were checked for their specificity against nucleotide databases at NCBI using Basic Local Alignment Search Tool (BLAST). Primers and probes were purchased from Eurogentec S.A. (Seraing, Belgium). For each primer/probe set, the amplification efficiency was tested, aiming to reach comparable efficiency of >90% (efficiency range from 97.7 to 99.7%). Primers and hydrolysis probes were diluted to 100 µM, using a stock solution with nuclease-free water (Life Technologies GmbH, Darmstadt, Germany). qRT-PCR was applied for the relative quantification of *CDKN2A* and *FGFR3*. For PCR, 0.5 µM of each primer and 0.25 µM of each probe were used. All quantitative reverse-transcription PCRs were performed in triplicates using the SuperScript® III Platinum® One-Step qRT-PCR kit (Invitrogen/Life Technologies, Darmstadt, Germany) according to the manufacturer’s instructions. Experiments were performed on a Stratagene Mx3005p (Agilent Technologies, Waldbronn, Germany) with 30 min at 50 °C and 2 min at 95 °C followed by 40 cycles of 15 s at 95 °C and 30 s at 60 °C. PCR amplification of each gene was performed in triplicates in each patient. Expression relative to CALM2 was determined using the 40-ΔCT method.

### Reanalysis of existing datasets

57 patients with MIBC (mean age 66 years, range 41–89) from the MDA cohort^45^ and 365 patients with MIBC (mean age 68 years, range 34–90 years) identified from the TCGA (The Cancer Genome Atlas) project served for outcome validation. Illumina array RNA expression data of the MDA cohort was downloaded from Gene Expression Omnibus (GSE48276).

TCGA RNA sequencing expression data (z-score normalized data) of *CDKN2A* and the drug target genes *FGFR3*, *AR*, *ESR1*, *ESR2*, *ERBB2*, *PDCD1* (PD1), *CD274* (PDL1) and *CTLA4* were downloaded from CBioPortal^47^. Furthermore the expression of TCGA molecular subtype determining genes *KRT5*, *KRT6A*, *KRT6B*, *KRT6C*, *KRT8*, *KRT14*, *KRT18*, *KRT20*, *UPK1A*, *UPK1B*, *UPK2*, *UPK3A*, *UPK3B*, *GATA3* and *FOXA1*, of the *CDKN2A* downstream target genes *CDK4 and RB1*, the copy number variation (CNV) data of *CDKN2A* and the annotated updated TCGA RNA expression subtypes^19^ were extracted.

### Statistics

Statistical analyses were performed using SAS JMP version 11.0 (SAS Institute, Cary, NC, USA) and Graphpad PRISM (Version 7.0; Graph Pad Software Inc., La Jolla, CA, USA). Cut-Off definitions were done by partitioning tests for decision trees to determine different *CDKN2A* expression groups. Student’s t-test and Chi^2^ test were used to compare for differences in the distribution of clinical parameters, TCGA subtypes and *CDKN2A* CNV data between the *CDKN2A* expression groups.

Kaplan Meier analyses were performed for DSS and RFS in the test cohort and for DSS and OS in the validation cohorts and were tested for significance using Log-Rank test.

Both in the test cohort and in the TCGA cohort *CDKN2A* expression was correlated with *FGFR3* expression using Spearman correlation. In the TCGA cohort *CDKN2A* expression was also correlated with the expression of *AR*, *ESR1*, *ESR2*, *ERBB2*, *PDCD1*, *CD274*, *CTLA4*, *KRT5*, *KRT6A*, *KRT6B*, *KRT6C*, *KRT8*, *KRT14*, *KRT18*, *KRT20*, *UPK1A*, *UPK1B*, *UPK2*, *UPK3A*, *UPK3B*, *GATA3*, *FOXA1*, *CDK4, RB1, E2F2* and *KI67*.

Student’s t-test was used to test for differences in gene expression in the *CDKN2A* expression groups in the test cohort and the TCGA cohort. For the analysis of *CDKN2A* expression according to *CDKN2A* copy number status and RNA-expression subtypes Kruskal-Wallis test with post hoc Dunn’s test for multiple comparisons was performed in the TCGA cohort. Graphs were designed with Graphpad Prism. P-values < 0.05 were deemed statistically significant.

### Ethical approval

All procedures performed in studies involving human participants were in accordance with the ethical standards of the institutional and/or national research committee and with the 1964 Helsinki declaration and its later amendments or comparable ethical standards. Due to its retrospective character, for this type of study formal consent is not required.

## Electronic supplementary material

Supplementary Information
