# Supplementary material for: CDKN2A as transcriptomic marker for muscle-invasive bladder cancer risk stratification and therapy decision-making
Source: Sci Rep. 2018 Sep 26;8:14383. doi: 10.1038/s41598-018-32569-x (PMC6158275; doi:10.1038/s41598-018-32569-x)
Supplement: Supplementary file 1 — Supplementary Information [file 41598_2018_32569_MOESM1_ESM.pdf]

CDKN2A as transcriptomic marker for muscle-invasive bladder cancer risk stratification and therapy decision-making

Thomas S. Worst[1]#, Cleo-Aron Weis[2]#, Robert Stöhr[3]*#, Simone Bertz[3]#, Markus Eckstein[3]#, Wolfgang Otto[4]#, Johannes Breyer[4]#, Arndt Hartmann[3]#, Christian Bolenz[5]#, Ralph M. Wirtz[6,7]# und Philipp Erben[1]#*

[1] Department of Urology, University Medical Center Mannheim, Theodor-Kutzer-Ufer 1-3, 68167 Mannheim, Germany, [2] Institute of Pathology, University Medical Center Mannheim, Theodor-Kutzer-Ufer 1-3, 68167 Mannheim, Germany, [3] Institute of Pathology, University of Erlangen-Nuremberg, Krankenhausstraße 8-10, 91054 Erlangen, Germany, [4] Department of Urology, University of Regensburg, Landshuter Straße 65, 93053 Regensburg, Germany, [5] Department of Urology, University of Ulm, Prittwitzstraße 43, 89075 Ulm, Germany, [6] STRATIFYER Molecular Pathology GmbH, Werthmannstraße 1, 50935 Cologne, Germany, [7] Institute of Pathology at the St Elisabeth Hospital Köln-Hohenlind, Werthmannstraße 1, 50935 Cologne, Germany
#on behalf of the BRIDGE Consortium

*** Corresponding Author**
Dr. med. Thomas Stefan Worst, University Medical Center Mannheim, Theodor-Kutzer-Ufer 1-3, 68167 Mannheim, Germany; Email: thomas.worst@medma.uni-heidelberg.de; Phone: +49 621 383 3245; Fax: +49 621 383 2032; ORCID: 0000-0001-5429-126X

Supplementary Table 2: Spearman correlation between *CDKN2A* and TCGA drug target genes and t-test of TCGA drug target genes in *CDKN2A* expression groups.

**correlated gene**	**Spearman**		**Student's t-test (p-value)**
	**ρ**	**p-value**	
FGFR3	-0.406	**<0.001**	**<0.001**
AR	-0.183	**0.004**	0.065
ESR1	0.069	0.187	0.201
ESR2	0.044	0.399	**<0.001**
ERBB2	-0.070	0.176	0.194
PDCD1	0.176	**<0.001**	0.589
CD274	0.327	**<0.001**	0.839
CTLA4	0.171	**<0.001**	0.632

Supplementary Table 2: Spearman correlation between *CDKN2A* and TCGA subtype specific genes and t-test of TCGA subtype specific genes in *CDKN2A* expression groups.

**correlated gene**	**Spearman**		**Student's t-test (p-value)**
	**ρ**	**p-value**	
KRT5	0.061	0.247	0.267
KRT6A	0.047	0.372	0.123
KRT6B	0.053	0.317	0.161
KRT6C	0.039	0.457	0.235
KRT8	-0.024	0.646	0.217
KRT14	0.013	0.804	0.580
KRT18	-0.009	0.853	0.093
KRT20	-0.043	0.409	**0.028**
UPK1A	-0.001	0.985	0.393
UPK1B	-0.024	0.651	0.508
UPK2	-0.033	0.528	0.255
UPK3A	<0.001	0.991	0.288
UPK3B	-0.008	0.885	0.119
GATA3	**-0.162**	**0.002**	**<0.001**
FOXA1	**-0.299**	**<0.001**	**<0.001**

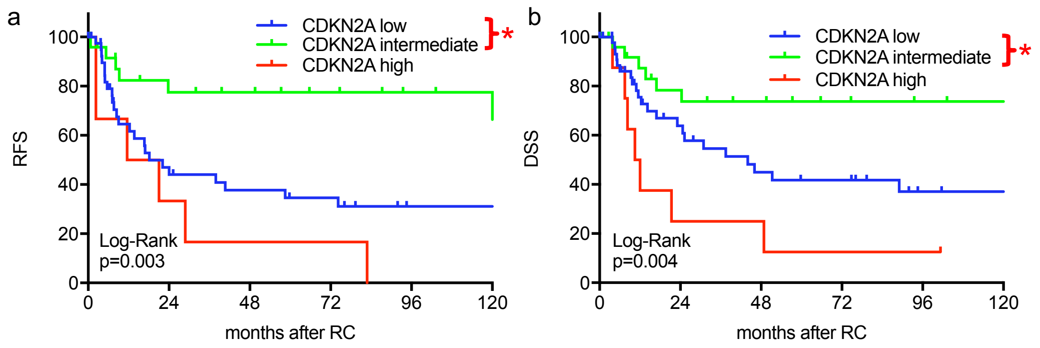

group	n	censored	event	median RFS
low	46	22	24	18.2
intermediate	26	19	7	n.r.
high	8	2	6	16.3

group	n	censored	event	median RFS
low	46	24	22	44.0
intermediate	26	19	7	n.r.
high	8	1	7	11.2

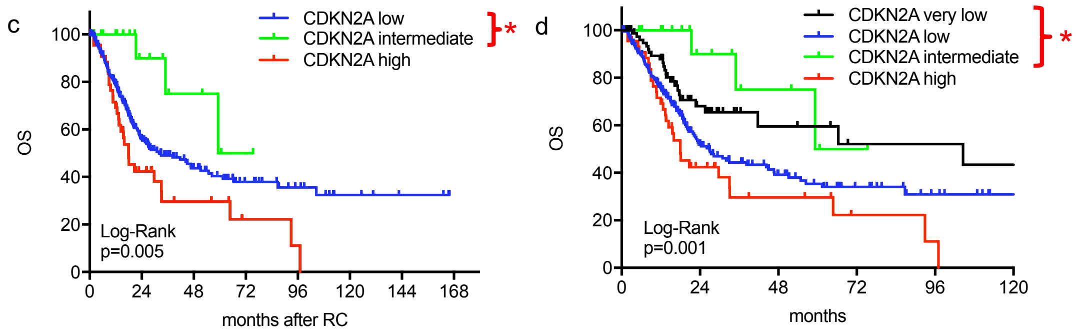

group	n	censored	event	median RFS
low	299	169	17	32.0
intermediate	20	17	3	67.3
high	46	17	29	18.0

group	n	censored	event	median RFS
very low	80	56	24	104.6
low	219	113	106	26.9
intermediate	20	17	3	67.3
high	46	17	29	18.0

Supplementary Figure 1: Further dissection of the test cohort (a+b) in three *CDKN2A* expression groups and of the TCGA cohort in 3 (c) or 4 (d) *CDKN2A* expression groups with the partition did not result in comparable sizes of the additional *CDKN2A* expression groups, being subgroups of the CDKN2A^{low} tumours from the initial analysis (indicated by red bracket and star). Though being different in cohort size, those patients with an intermediate expression had a better prognosis compared to those with a low expression.

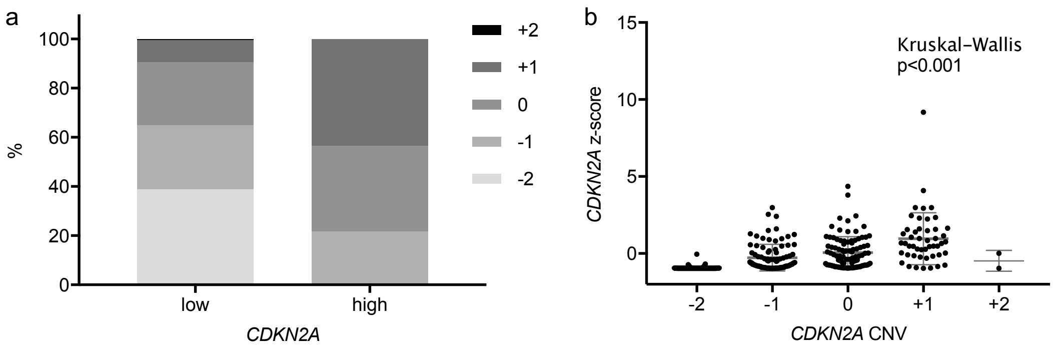

Supplementary Figure 2: a) In the TCGA cohort none of the tumours in the *CDKN2A*^{high} had a homozygous deletion of *CDKN2A, whilst in the CDKN2A*^{low} *group these were 38.9%.* b) The *CDKN2A* expression significantly increased with the copy number. Only two tumours had a gene amplification of +2.

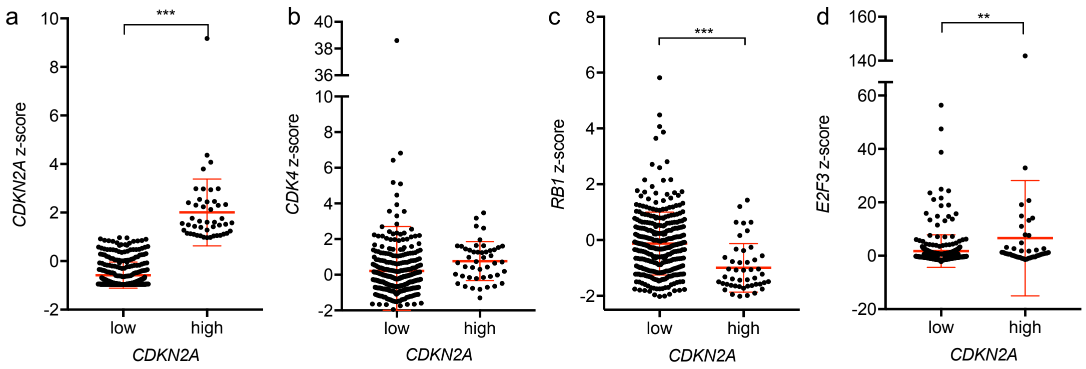

Supplementary Figure 3: a) *CDKN2A* expression in the four different groups. b-d) The downstream targets *CDK4, RB1* and *E2F3* in showed a differing expression the *CDKN2A* expression groups. (*p<0.05; **p<0.01; ***p<0.001)
